# Regulation of *Trypanosoma brucei* Acetyl Coenzyme A Carboxylase by Environmental Lipids

**DOI:** 10.1128/mSphere.00164-18

**Published:** 2018-07-11

**Authors:** Sunayan S. Ray, Christina L. Wilkinson, Kimberly S. Paul

**Affiliations:** a Department of Genetics & Biochemistry, Clemson University, Clemson, South Carolina, USA; b Eukaryotic Pathogens Innovation Center (EPIC), Clemson University, Clemson, South Carolina, USA; University of Georgia

**Keywords:** *Trypanosoma brucei*, acetyl-CoA carboxylase, enzyme regulation, fatty acids, lipids, parasitology, protein phosphorylation

## Abstract

To satisfy its fatty acid needs, the extracellular eukaryotic parasite Trypanosoma brucei relies on two mechanisms: uptake of fatty acids from the host and *de novo* synthesis. We hypothesized that T. brucei modulates fatty acid synthesis in response to environmental lipid availability. The first committed step in fatty acid synthesis is catalyzed by acetyl coenzyme A (acetyl-CoA) carboxylase (ACC) and serves as a key regulatory point in other organisms. To test our hypothesis, T. brucei mammalian bloodstream and insect procyclic forms were grown in low-, normal-, or high-lipid media and the effect on T. brucei ACC (TbACC) mRNA, protein, and enzymatic activity was examined. In bloodstream form T. brucei, media lipids had no effect on TbACC expression or activity. In procyclic form T. brucei, we detected no change in TbACC mRNA levels but observed 2.7-fold-lower TbACC protein levels and 37% lower TbACC activity in high-lipid media than in low-lipid media. Supplementation of low-lipid media with the fatty acid stearate mimicked the effect of high lipid levels on TbACC activity. In procyclic forms, TbACC phosphorylation also increased 3.9-fold in high-lipid media compared to low-lipid media. Phosphatase treatment of TbACC increased activity, confirming that phosphorylation represented an inhibitory modification. Together, these results demonstrate a procyclic-form-specific environmental lipid response pathway that regulates TbACC posttranscriptionally, through changes in protein expression and phosphorylation. We propose that this environmental response pathway enables procyclic-form T. brucei to monitor the host lipid supply and downregulate fatty acid synthesis when host lipids are abundant and upregulate fatty acid synthesis when host lipids become scarce.

**IMPORTANCE** Trypanosoma brucei is a eukaryotic parasite that causes African sleeping sickness. T. brucei is transmitted by the blood-sucking tsetse fly. In order to adapt to its two very different hosts, T. brucei must sense the host environment and alter its metabolism to maximize utilization of host resources and minimize expenditure of its own resources. One key nutrient class is represented by fatty acids, which the parasite can either take from the host or make themselves. Our work describes a novel environmental regulatory pathway for fatty acid synthesis where the parasite turns off fatty acid synthesis when environmental lipids are abundant and turns on synthesis when the lipid supply is scarce. This pathway was observed in the tsetse midgut form but not the mammalian bloodstream form. However, pharmacological activation of this pathway in the bloodstream form to turn fatty acid synthesis off may be a promising new avenue for sleeping sickness drug discovery.

## Abstract (precis)

Trypanosoma brucei is a eukaryotic parasite that causes African sleeping sickness. T. brucei is transmitted by the blood-sucking tsetse fly. In order to adapt to its two very different hosts, T. brucei must sense the host environment and alter its metabolism to maximize utilization of host resources and minimize expenditure of its own resources. One key nutrient class is represented by fatty acids, which the parasite can either take from the host or make themselves. Our work describes a novel environmental regulatory pathway for fatty acid synthesis where the parasite turns off fatty acid synthesis when environmental lipids are abundant and turns on synthesis when the lipid supply is scarce. This pathway was observed in the tsetse midgut form but not the mammalian bloodstream form. However, pharmacological activation of this pathway in the bloodstream form to turn fatty acid synthesis off may be a promising new avenue for sleeping sickness drug discovery.

## INTRODUCTION

Human and animal African trypanosomiasis is primarily caused by Trypanosoma brucei spp., eukaryotic parasites transmitted by the blood-feeding tsetse fly (*Glossina* spp.). In its mammalian host, the bloodstream form (BF) of the parasite exists extracellularly in the bloodstream, lymph, skin, adipose interstitial spaces, and cerebrospinal fluid. When the tsetse fly feeds on an infected mammal, T. brucei parasites enter the tsetse fly midgut, where they differentiate into procyclic forms (PFs). These PFs eventually migrate to the salivary glands, where they undergo development to infectious metacyclic forms primed for transmission to the next mammalian host.

During its life cycle, T. brucei encounters multiple host microenvironments that differ and that can vary in their availability of nutrients, including lipids. For example, the parasite experiences an ~400-fold drop in lipid availability when it crosses the blood-brain barrier and enters the cerebrospinal fluid of its mammalian hosts (1). Alternatively, the parasite may experience a dramatic increase in lipid availability when it colonizes the adipose tissue (2). To successfully adapt to its host environments, T. brucei must sense such changes in nutrient availability and respond by undergoing developmental, behavioral, and/or metabolic changes (3, 4). One important class of lipids is fatty acids. In addition to being main structural components of membrane phospholipids, fatty acids serve as anchors for membrane proteins through direct acylation of the protein or via a more complex glycosylphosphatidylinositol (GPI) anchor. In T. brucei, GPI-anchored surface proteins play critical roles in protecting the parasite from host defenses (4, 5). A sufficient supply of fatty acids is needed to maintain surface coat integrity as well as membrane lipid homeostasis. To satisfy its fatty acid needs, T. brucei relies on two mechanisms: uptake of environmental fatty acids from the host and *de novo* synthesis. T. brucei readily takes fatty acids from its host (6, 7). However, if exogenous fatty acids are limiting, T. brucei can synthesize fatty acids *de novo* through one of two pathways: an endoplasmic reticulum (ER)-localized fatty acid elongase (ELO) pathway and a mitochondrial type II fatty acid synthesis pathway (8–10).

Fatty acid synthesis requires malonyl coenzyme A (malonyl-CoA) as a substrate, which serves as the two-carbon donor for acyl chain elongation. Acetyl coenzyme A (acetyl-CoA) carboxylase (ACC) catalyzes the synthesis of malonyl-CoA from acetyl-CoA in what constitutes the first committed step in fatty acid synthesis. ACC is a member of the biotin carboxylase family, whose members use a biotin prosthetic group to transfer a carboxyl group from a bicarbonate donor to an acceptor acetyl-CoA, forming the malonyl-CoA product (11). Because it catalyzes the first committed step in fatty acid synthesis, ACC serves as a key control point for regulating this pathway, where it is subjected to multiple modes of transcriptional and posttranslational regulation, including phosphorylation (12). Although ACC is a target of multiple kinases, the best-characterized ACC kinase is AMP-activated protein kinase (AMPK), a global regulator of cellular energy metabolism activated by the presence of a low cellular energy charge (13). AMPK phosphorylation of ACC inhibits its activity, turning off fatty acid synthesis to conserve energy during starvation or stress (14).

We previously characterized T. brucei ACC (TbACC) and found that it is well conserved among trypanosomes at the amino acid level (~60% identity) but diverges from fungal and mammalian ACCs (~30% identity) (15). RNA interference (RNAi) knockdown of TbACC mRNA in PFs caused a growth defect only when levels of environmental lipids were low (15), suggesting that fatty acid synthesis was especially important when the exogenous fatty acid supply was limiting. This idea is supported by prior data showing that T. brucei upregulated fatty acid synthesis when grown under lipid-poor conditions (9, 16).

Given these observations, we hypothesized that T. brucei parasites can sense their environmental lipid supply and respond accordingly by modulating their fatty acid synthesis pathways. Given the central role that TbACC likely plays in T. brucei fatty acid metabolism and its multiple modes of regulation in other organisms, we examined the possibility that TbACC is regulated in response to environmental lipids as part of this environmental sensing pathway.

We investigated the effect of growth in low-, normal-, and high-lipid media on TbACC mRNA, protein, and activity. While TbACC mRNA levels did not change in response to altered environmental lipid levels, we observed increased TbACC protein levels and TbACC enzyme activity after growth under low-lipid conditions. We found that TbACC was phosphorylated under high-lipid conditions and demonstrated that phosphorylation inhibited TbACC activity. Finally, we determined that regulation of TbACC by environmental lipids appears to be stage specific, occurring only in PFs and not in BFs.

## RESULTS

### Effect of environmental lipids on TbACC mRNA and protein levels.

To examine the regulation of TbACC at the level of transcription and/or mRNA stability, we used quantitative reverse transcriptase PCR (qRT-PCR) to quantify changes in steady-state TbACC mRNA levels in response to different environmental lipid conditions. (For details of the qRT-PCR methods used, see Text S1 in the supplemental material.) BF and PF cells were grown for 72 h in low-, normal-, and high-lipid media, conditions that were created by altering the serum content in the media (Table 1). Total RNA was isolated and used to generate cDNA as the template for qRT-PCR using primer sets for TbACC and actin as the internal control. qRT-PCR revealed similar TbACC mRNA levels in BFs and PFs, with no significant difference between cells grown under low-, normal-, and high-lipid conditions (see Fig. S1 in the supplemental material).

10.1128/mSphere.00164-18.1 TEXT S1  Supplemental information. Download TEXT S1, DOCX file, 0.2 MB. Copyright © 2018 Ray et al. 2018 Ray et al. This content is distributed under the terms of the Creative Commons Attribution 4.0 International license.

10.1128/mSphere.00164-18.2 FIG S1  Environmental lipids do not affect TbACC mRNA levels in PF and BF. BF and PF WT cells were grown in low-, normal-, and high-lipid media (see Table 1 for media formulations) to the mid-log phase (~3 days). TbACC mRNA levels were assessed by qRT-PCR, normalized to β-actin, and then normalized to the normal-lipid condition. Data show the normalized means ± SEM of results from three independent experiments. No significant differences (*P* ≤ 0.05) were found (two-tailed Student’s *t* test). Download FIG S1, TIF file, 0.6 MB. Copyright © 2018 Ray et al. 2018 Ray et al. This content is distributed under the terms of the Creative Commons Attribution 4.0 International license.

**TABLE 1:** Formulations for low-, normal-, and high-lipid media

T. brucei developmental stage^a^	Media condition	Serum components added to media			% final serum lipid equivalents (fold concentration of normal-lipid media)^b^
		% Delipidated fetal bovine serum (FBS)^b^	% FBS^b^	% Serum Plus^b^	
BF	Low	10	—^c^	10	4 (~0.2×)
	Normal	—	10	10	12 (1×)
	High	—	20	10	22 (~2×)
PF	Low	10	—	—	2 (0.2×)
	Normal	—	10	—	10 (1×)
	High	—	20	—	20 (2×)

a BF, bloodstream form; PF, procyclic form.

b % vol/vol.

c Dash indicates no addition.

**FIG 1:**
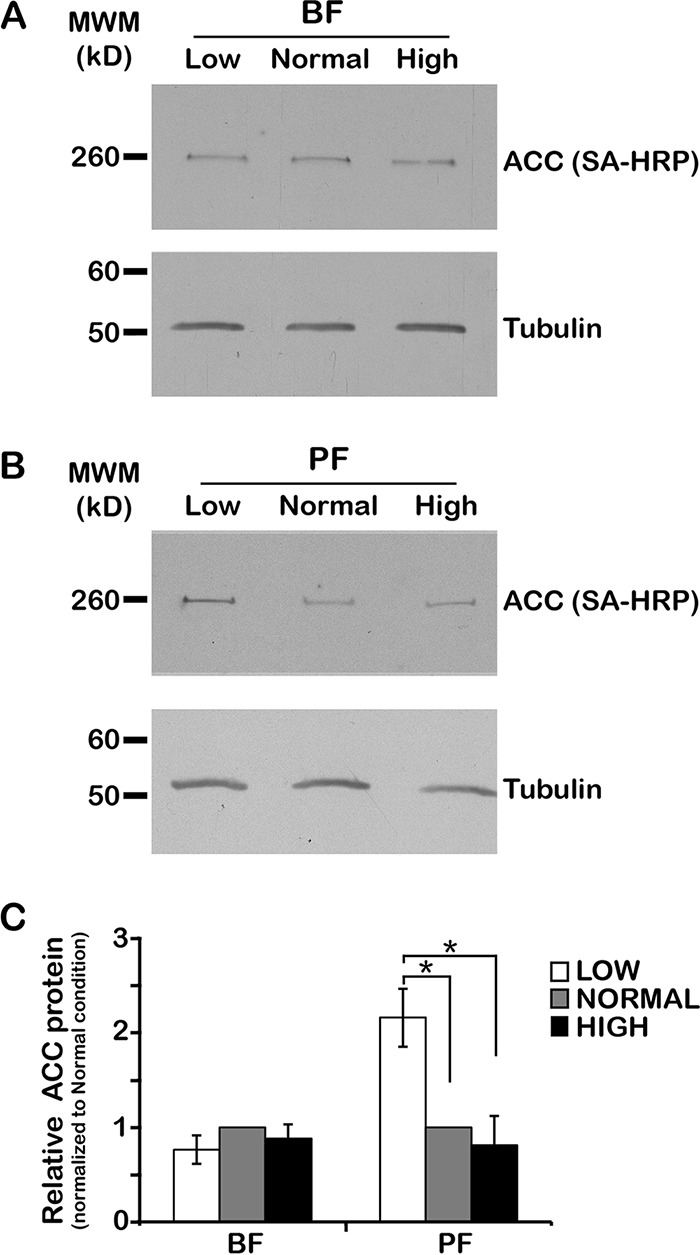
Environmental lipids affect TbACC protein levels in PF but not BF. (A and B) BF (A) and PF (B) wild-type (WT) cells were grown in low-, normal-, and high-lipid media to the mid-logarithmic (mid-log) phase (~3 days). Lysates prepared in the presence of a phosphatase inhibitor cocktail were resolved by the use of SDS-PAGE (10 µg total protein/lane) and transferred to nitrocellulose. TbACC was detected by SA-HRP blotting (A and B, top panels), and the same blots were reprobed for tubulin as a loading control (bottom panels). Representative blots from three independent experiments are shown. MWM, molecular weight marker. (C) Densitometric quantification of the results from the three independent experiments described in the panel A and B legends. The TbACC signal was normalized to the tubulin loading control. Means ± standard errors of the means (SEM) are shown. *, *P* ≤ 0.05 (two-tailed Student’s *t* test).

To investigate the possibility of TbACC regulation at the level of translation and protein stability, we examined the effect of environmental lipids on TbACC protein levels by Western blotting with streptavidin conjugated to horseradish peroxidase (SA-HRP), which binds to the biotin prosthetic group of ACC (15, 17, 18). The predicted size of the TbACC protein is 243 kDa, which allows it to be readily distinguished from the single other biotinylated protein encoded in the T. brucei genome, the 71-kDa α subunit of 3-methylcrotonyl-CoA carboxylase (TriTrypDB gene identifier [ID]: Tb927.8.6970), an enzyme involved in amino acid degradation. Cell lysates prepared from BFs and PFs grown in low-, normal-, and high-lipid media for 72 h were resolved by sodium dodecyl sulfate-polyacrylamide gel electrophoresis (SDS-PAGE), transferred to nitrocellulose, and probed for TbACC by SA-HRP blotting. In BFs, TbACC protein levels showed no significant difference in cells grown in low, normal, or high lipid media (Fig. 1A and C). In PFs, however, TbACC protein levels changed in response to environmental lipid levels. Growth in low-lipid media led to higher TbACC protein levels, and growth in high-lipid media led to lower TbACC protein levels, with a significant (*P* = 0.019) 2.7-fold difference in TbACC levels in cells grown in low-lipid versus high-lipid medium conditions, and a more modest 2.2-fold increase (*P* = 0.019) in TbACC protein was observed in low-lipid media compared to normal media (Fig. 1B and C). This same general pattern of TbACC protein levels was observed under conditions of TbACC depletion by RNA interference, though the overall levels of TbACC were very low and the differences between the different growth media were not statistically significant (Fig. S2).

10.1128/mSphere.00164-18.3 FIG S2  Environmental lipids had a minor effect on TbACC protein levels under ACC RNAi conditions. (A) PF ACC RNAi cells were grown for 7 days in normal media under RNAi induction conditions (media supplemented with 1 µg/ml tetracycline) or solvent control conditions (media supplemented with 0.1% dimethyl sulfoxide [DMSO]), with passage every 2 to 3 days. The induced and uninduced control cultures were divided into low-, normal-, and high-lipid media cultures and then grown for an additional 2 days to the mid-log phase, for a total RNAi induction period of 9 days. Lysates prepared in the presence of a phosphatase inhibitor cocktail were resolved by the use of SDS-PAGE (10 µg total protein/lane) and transferred to nitrocellulose. Blots were cut, and the top half was probed for TbACC by SA-HRP blotting (top panel), and the lower half was probed for tubulin as a loading control (bottom panel). A representative blot from three independent experiments is shown. The asterisk indicates a nonspecific SA-HRP cross-reacting band. (B) Densitometric quantification of results from three independent experiments performed as described in the panel A legend. TbACC signal was normalized to the tubulin loading control and then normalized to the uninduced normal-media condition. Means ± SEM are shown. *, *P* ≤ 0.05 (two-tailed Student’s *t* test). Download FIG S2, TIF file, 1 MB. Copyright © 2018 Ray et al. 2018 Ray et al. This content is distributed under the terms of the Creative Commons Attribution 4.0 International license.

### Effect of environmental lipids on TbACC activity.

**FIG 2:**
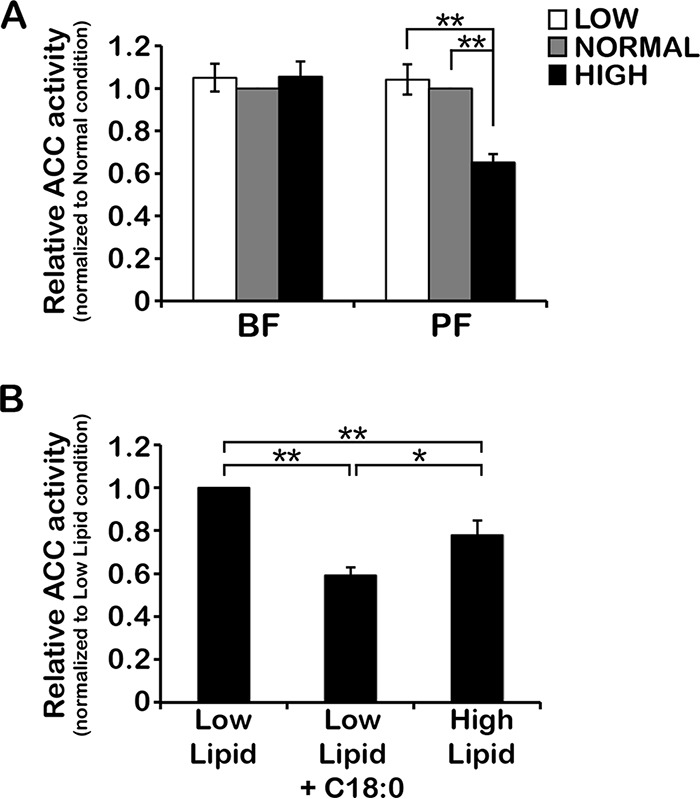
Environmental lipids affect TbACC activity in PF but not BF. (A) BF and PF WT cells were grown under low-, normal-, and high-lipid conditions to the mid-log phase (~3 days). Lysates prepared in the presence of phosphatase inhibitor cocktail (equal total levels of protein, 2 to 10 µg) were assayed for TbACC activity by measuring incorporation of [^14^C]NaHCO_3_ into the acid-resistant malonyl-CoA product in the presence of ATP and acetyl-CoA. Values were first normalized to the no-ATP negative control before averaging was performed. Average values were then expressed relative to that of normal-lipid media. Means ± SEM of results from three independent experiments performed in pentuplicate are shown. **, *P* ≤ 0.005 (two-tailed Student’s *t* test). (B) PF WT cells grown under low-lipid conditions to the mid-log phase (~2 days) were subdivided into three cultures and grown for an additional 24 h, with one culture maintained in low-lipid media (Low Lipid), one supplemented with a final concentration of 20% FBS (High Lipid), and one supplemented with 35 µM stearate fatty acid (Low Lipid + C18:0). Hypotonic lysates were prepared and assayed for TbACC activity as described above. Values were normalized to the no-ATP control before averaging was performed, and averaged values are expressed relative to the low-lipid condition. Means ± SEM of results from three independent experiments are shown. *, *P* ≤ 0.05; **, *P* ≤ 0.005 (two-tailed Student’s *t* test).

To examine the effect of environmental lipids on TbACC activity, hypotonic lysates were prepared from BF and PF cells grown in low-, normal-, and high-lipid media for 72 h and were then passed through a small gel filtration column to exchange the lysate into enzyme assay buffer and remove endogenous substrates. TbACC activity was assayed in the gel-filtered lysates by measuring the incorporation of ^14^CO_2_ from NaH^14^CO_3_ in the presence of acetyl-CoA and ATP into the acid-resistant product [^14^C]malonyl-CoA, which was collected onto filter paper and quantified by scintillation counting. In BFs, TbACC activity showed no significant differences between cells grown in low-, normal-, or high-lipid media (Fig. 2A), consistent with the lack of change seen in TbACC protein levels in BFs in response to altering the levels of the lipids in the media. In PFs, no difference in TbACC activity was seen between cells grown in low and normal-lipid media, but growth in high-lipid media resulted in significant ~35% (*P* = 5.2 × 10^−10^) and ~37% (*P* = 2.9 × 10^−5^) decreases in TbACC activity compared to growth in normal-lipid and low-lipid media, respectively (Fig. 2A). The observed changes in TbACC activity in PFs were consistent with the observed changes in PF TbACC protein levels in the different lipid media.

To confirm that the observed changes in TbACC activity in PFs grown in high-lipid media were due to the lipid component of the media, we determined if addition of exogenous fatty acids would affect TbACC activity. PFs were first passaged into low-lipid media and grown to the mid-logarithmic (mid-log) stage. The culture was then divided into three subcultures that were (i) maintained in low-lipid media, (ii) supplemented with fetal bovine serum (FBS) to mimic the high-lipid media, or (iii) supplemented with 35 µM stearic acid (C18:0), an abundant fatty acid, at a concentration similar to that in mammalian serum and sufficient to rescue the growth of PFs with reduced ELO pathway activity in low-lipid media (9, 19). After 24 h under the new media conditions, TbACC activity was assayed as described above. Compared to cells maintained in low-lipid media, addition of serum (high lipid) reduced TbACC activity by 22% (*P* = 0.0022) compared to the low-lipid condition (Fig. 2B), consistent with the result shown in Fig. 2A. Addition of 35 µM fatty acid stearate (C18:0) caused an effect similar to that seen with serum addition, significantly (*P* = 1.2 × 10^−12^) decreasing TbACC activity by 41% (Fig. 2B).

### Effect of environmental lipids on phosphorylation of ACC.

**FIG 3:**
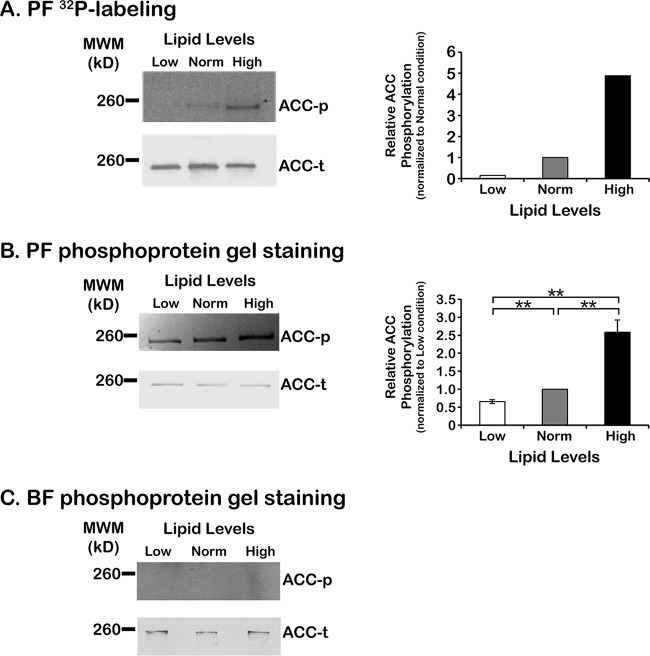
Effect of environmental lipids on PF TbACC phosphorylation. (A) PF TbACC-myc cells were grown in normal-lipid media to the mid-log phase (~2 days). Cells were harvested and then incubated with 1 to 2 mCi [^32^P]orthophosphate in low (Low)-, normal (Norm)-, and high (High)-lipid phosphate-free media for 16 h. TbACC-myc immunoprecipitates were resolved by the use of SDS-PAGE, transferred to nitrocellulose, and assessed by autoradiography (top left panel). An identically loaded blot was prepared in parallel and probed for total TbACC by SA-HRP blotting (bottom left panel). Densitometric analysis of the blot is shown in the right panel. TbACC-myc phosphorylation values were normalized to total TbACC loaded levels. This experiment was performed once. (B) PF TbACC-myc cells were grown in low-, normal-, and high-lipid media to the mid-log phase (~3 days). TbACC-myc immunoprecipitates were resolved by the use of 10% SDS-PAGE, and phosphorylated TbACC-myc was detected by phosphoprotein gel staining and imaging under UV (upper left panel, ACC-p). Identically loaded gels prepared in parallel were transferred to nitrocellulose and probed for total TbACC by SA-HRP blotting (lower left panel, ACC-t). A representative gel and blot from three independent experiments are shown. Densitometric analysis of three independent experiments is shown in the right panel. PF TbACC-myc phosphorylation values (ACC-p) were normalized to total TbACC-myc loaded levels (ACC-t). Means ± SEM are shown. **, *P* ≤ 0.005 (two-tailed Student’s *t* test). (C) BF TbACC-myc cells were grown in low-, normal-, and high-lipid media to the mid-log phase (~3 days). TbACC-myc immunoprecipitates were resolved by the use of SDS-PAGE and assessed for phosphorylated TbACC (ACC-p) by phosphoprotein gel staining (top panel) and total TbACC (ACC-t) by SA-HRP blotting (bottom panel) as described for panel B. No densitometry data are shown due to a lack of phosphorylated TbACC.

ACC is regulated by phosphorylation in a diverse array of organisms, from yeast to humans. TbACC was identified as a phosphoprotein by proteomics (20), and we showed that treatment of PF and BF lysates with (–)-epigallocatechin gallate (EGCG), a bioactive compound in green tea, resulted in increased TbACC phosphorylation and decreased TbACC activity in cell lysates (21). Taken together, these observations suggested that TbACC may be subject to phosphoregulation in response to some stimuli. Thus, we examined whether TbACC is differentially phosphorylated in intact cells in response to environmental lipids. To do this, we exploited a previously published PF cell line in which one of the endogenous TbACC genomic loci was modified with a C-terminal c-myc epitope tag (PF ACC-myc) (15), allowing expression of myc-tagged TbACC under the control of its endogenous promoter. PF ACC-myc cells were grown in low-, normal-, and high-lipid media for 48 h and then metabolically labeled in low-phosphate media with [^32^P]orthophosphate as the main source of phosphate. TbACC-myc was isolated from hypotonic lysates via immunoprecipitation with c-myc antibody conjugated to agarose beads, and immunoprecipitates were separated by SDS-PAGE, transferred to nitrocellulose, and subjected to autoradiography. Total TbACC protein levels were determined by SA-HRP blotting after the ^32^P signal decayed to background. As we observed previously (21), [^32^P]orthophosphate labeling of TbACC-myc immunoprecipitates demonstrated that TbACC was phosphorylated in PFs (Fig. 3A, upper left panel). However, phosphorylation of TbACC varied in response to different lipid levels in the media. Densitometric quantification showed that growth in low-lipid media resulted in an 85% decrease in TbACC-myc phosphorylation compared to normal media, while growth in high-lipid media resulted in 4.9-fold and 32-fold increases in TbACC-myc phosphorylation compared to normal-lipid and low-lipid media, respectively (Fig. 3A, right panel).

We confirmed and further explored the effect of media lipids on TbACC phosphorylation using a nonradioactive phosphoprotein staining method. PF ACC-myc cells were grown in low-, normal-, and high-lipid media for 48 h, and TbACC-myc was purified from hypotonic lysates by immunoprecipitation with anti-myc agarose beads as described above. TbACC-myc immunoprecipitates were resolved by the use of SDS-PAGE, and then the gel was stained with Pro-Q Diamond stain, which specifically stains phosphoproteins in acrylamide gels. To determine total TbACC levels, gels prepared in parallel were transferred to nitrocellulose and probed by SA-HRP Western blotting. As with the metabolic [^32^P]orthophosphate labeling, PF TbACC-myc in cells grown under different lipid media conditions showed different levels of phosphorylation by phosphoprotein gel staining (Fig. 3B, upper left panel). Low-lipid media resulted in a significant (*P* = 0.0012) ~34% decrease in TbACC phosphorylation compared to normal-lipid media. In contrast, high-lipid media induced the highest level of TbACC phosphorylation with significant 2.6-fold (*P* = 0.0092) and 3.9-fold (*P* = 0.0047) increases over that in normal-lipid and low-lipid conditions, respectively (Fig. 3B, right panel). These changes in PF TbACC phosphorylation in low- and high-lipid media are consistent in scale with the modulation of TbACC activity under the same growth conditions.

Previously, we observed undetectable levels of phosphorylation in BFs grown in normal media (21). To examine if TbACC phosphorylation in BFs was affected by media lipid levels, we cultured BF TbACC-myc cells in low-, normal-, and high-lipid media and analyzed TbACC-myc immunoprecipitates for phosphorylation as described above. Phosphoprotein gel staining of BF TbACC-myc immunoprecipitates revealed no detectable phosphorylation and no change in phosphorylation of TbACC-myc under any growth condition in BFs (Fig. 3C). This lack of apparent phosphoregulation of BF TbACC under the different lipid media conditions mirrors the prior observed absence of change in BF TbACC protein and activity levels.

### Phosphorylation reduces the activity of TbACC-myc.

**FIG 4:**
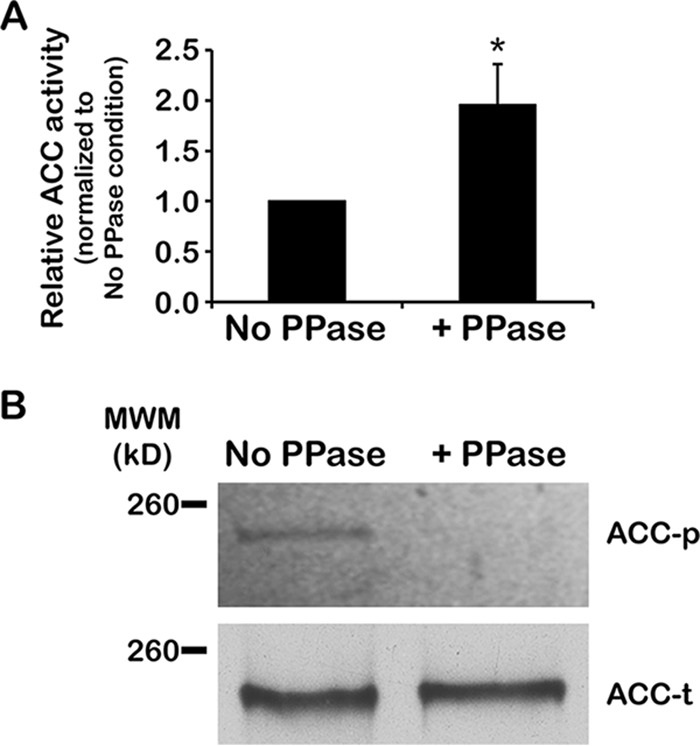
Phosphorylation of TbACC reduces activity. (A) PF TbACC-myc cells were grown in normal media to the mid-log phase (~3 days). TbACC-myc was immunoprecipitated from lysates and directly treated on-bead with 400 U of Lambda phosphatase (+PPase) or was subjected to mock treatment as a control (No PPase). Phosphatase- and mock-treated TbACC-myc was directly assayed on-bead for ACC activity. Values were first normalized to the no-ATP negative control before averaging was performed. Average values were then expressed relative to that of normal-lipid media. Means ± SEM of results from three independent experiments are shown. *, *P* ≤ 0.05 (two-tailed Student’s *t* test). (B) To confirm dephosphorylation, results of phosphatase- and mock-treated TbACC-myc pulldown experiments were resolved by the use of SDS-PAGE and assessed for phosphorylation by phosphoprotein gel staining (upper panel, ACC-p). An identically loaded gel was prepared in parallel, transferred to nitrocellulose, and probed for total TbACC by SA-HRP blotting (lower panel, ACC-t). The image was digitally processed to enable better visualization of the bands (see Materials and Methods). This experiment was performed once.

Previously, we observed a negative correlation between TbACC phosphorylation and TbACC activity in cell lysates (21). To directly determine the effect of phosphorylation on TbACC activity, PF TbACC-myc cells were grown in high-lipid media for 72 h, and TbACC-myc was isolated with anti-myc agarose beads. Bound TbACC-myc was treated on-bead with bacteriophage Lambda protein phosphatase or left untreated as a control. The TbACC-myc bound beads were exchanged into biotin carboxylase buffer and assayed on-bead for TbACC activity as described above. Phosphatase treatment of TbACC-myc resulted in a significant (*P* = 0.046) 1.9-fold increase in TbACC-myc ACC activity compared to the untreated control, indicating that altered phosphorylation affects TbACC activity and that phosphorylation is an inhibitory modification (Fig. 4A). To confirm dephosphorylation of TbACC-myc by Lambda phosphatase, treated and untreated TbACC-myc immunoprecipitates were assessed by SDS-PAGE and phosphoprotein gel staining as described above. Pro-Q Diamond phosphoprotein gel staining showed reduced phosphostaining of the phosphatase-treated TbACC-myc, indicating successful dephosphorylation (Fig. 4B; see also Fig. S3).

10.1128/mSphere.00164-18.4 FIG S3  Lambda phosphatase treatment dephosphorylates TbACC. PF TbACC-myc cells were grown in normal media to the mid-log phase (~3 days). TbACC-myc was immunoprecipitated from lysates and treated on-bead with 400 U of Lambda phosphatase (+PPase) or subjected to mock treatment as a control (No PPase). Results of phosphatase- and mock-treated TbACC-myc pulldown experiments were resolved by the use of SDS-PAGE and assessed for phosphorylation by phosphoprotein gel staining (upper panel, ACC-p). An identically loaded gel was prepared in parallel, transferred to nitrocellulose, and probed for total TbACC by SA-HRP blotting (lower panel, ACC-t). This experiment was performed once. Download FIG S3, TIF file, 0.3 MB. Copyright © 2018 Ray et al. 2018 Ray et al. This content is distributed under the terms of the Creative Commons Attribution 4.0 International license.

## DISCUSSION

Our findings showed that T. brucei responded to altered environmental lipid availability by posttranscriptional modulation of TbACC activity. This environmental response pathway appears to be specific to PFs, as no TbACC regulation was observed in BFs in response to different medium lipid levels. One possible explanation is that PFs use this environmental lipid response pathway to adapt to the available resources in the different tsetse fly tissues that it encounters during its life cycle (4), while BFs may not require this response under the relatively constant conditions in the bloodstream. Additionally, BFs may require TbACC to be constitutively activated to meet the fatty acid synthesis requirements needed to secure sufficient myristate for the VSG GPI anchors (8).

Although transcriptional regulation of ACC is nearly universal in all other organisms studied to date (22), TbACC exhibited no change in steady-state mRNA levels in response to altered lipid availability, suggesting a lack of transcriptional control. TbACC is therefore unusual in this aspect of its regulation compared to other ACCs. However, the lack of transcriptional regulation is not unexpected, as T. brucei is known to undergo minimal transcriptional regulation due to polycistronic transcription and the absence of classical RNA polymerase (Pol) II promoters (reviewed in references 23 and 24). Therefore, the observed changes in TbACC protein levels likely occurred posttranscriptionally. T. brucei is known to regulate gene expression at the level of mRNA stability (25), but the unchanged TbACC mRNA levels argue against this type of regulation.

One possible mode of posttranscriptional TbACC regulation could involve changes in the efficiency of TbACC mRNA translation mediated directly by altering mRNA structure or indirectly through the action of mRNA binding proteins (24). For example, the human epidermal growth factor receptor 2 (HER2) oncogene upregulates ACC expression in breast cancer cells by inducing translational derepression of ACC mRNAs (26). In the trypanosomatid Leishmania amazonensis, heat shock conferred selective translation of the HSP83 heat shock protein through a thermally responsive structural element in the HSP83 3′ untranslated region (3′-UTR) (27). In T. brucei, the TbZFP3 mRNA binding protein positively regulated expression of specific procyclins by increasing association with the translation machinery (28). A second possible mode of regulation of TbACC protein levels could involve changes in TbACC turnover via the ubiquitin/proteasome pathway (29, 30). For example, ACC protein levels in tumor cells were upregulated through the overexpression of proteins that directly bind ACC and prevent its degradation (31, 32). In T. brucei, heat shock stabilized the ZC3H11 stress response protein by increasing phosphorylation of a site(s) on ZC3H11 that inhibits ubiquitination and subsequent turnover by the proteasome (30).

In addition to changes in TbACC protein levels, we showed that altered environmental lipid levels lead to changes in TbACC phosphorylation. Our findings are consistent with our prior data showing that basal TbACC phosphorylation is higher in PFs and essentially undetectable in BFs (21), though BF TbACC was capable of being phosphorylated in lysates treated with EGCG. In addition, Urbaniak et al. identified TbACC as a phosphorylated protein in both BFs and PFs in a phosphoproteomics study (20), though our results differed from those of Urbaniak et al. with respect to the relative levels of TbACC phosphorylation observed in BFs and PFs. Using phosphoprotein gel staining, we readily detected TbACC phosphorylation in PFs but not BFs grown in normal-lipid media. In contrast, Urbaniak et al. detected S5- and S1999/S2001-containing phosphopeptides at similar levels (±1.3-fold) in PFs and BFs (20). Methodological differences and the higher sensitivity of mass spectrometry could account for the differences in our results. However, comparisons of ratios of the TbACC phosphosites were possible in only one or two of the four parallel phosphoproteomic experiments of Urbaniak et al. (20), and no TbACC phosphorylation in BFs was detected in an earlier phosphoproteomic study (33), suggesting a relatively low overall abundance of TbACC phosphopeptides under normal growth conditions. Nevertheless, our data demonstrated that growth in high-lipid media was sufficient to induce an increase in TbACC phosphorylation in PFs detectable by phosphoprotein staining, suggesting a robust change in TbACC phosphorylation in response to environmental lipids.

Comparison to known regulatory phosphosites on mammalian and yeast ACCs revealed no homologous sites in TbACC, an unsurprising result given the lack of sequence conservation at these sites (see Text S1 and Fig. S4 in the supplemental material) (34). *In silico* analysis using phosphoprediction algorithms trained on mammalian and yeast phosphosites revealed that 10 sites in TbACC scored highly by both algorithms (see Text S1 and Table S1 in the supplemental material) and that only 1 (S1046) is located in a region of TbACC similar to a known ACC regulatory phosphosite: the linker region between the biotin carboxyl carrier and carboxyltransferase domains (Fig. S4) (12, 35, 36). Of the three phosphorylated Ser residues on TbACC identified by mass spectrometry, S5 is located in a region similar to that of the N-terminal regulatory phosphosites S78/S80 in mammalian ACCs (Fig. S4), while the location of S1999/S2001 sites is novel, with no analog in other ACCs. Of the three detected TbACC phosphosites, only S2001 was scored highly by both *in silico* prediction algorithms (Table S1), while the other two sites were predicted by only one of the algorithms with moderate confidence. This discordance suggests the limits of the use of existing phosphoprediction algorithms in identifying bona fide phosphosites in T. brucei proteins, likely due to the evolutionary divergence of T. brucei confounding algorithms trained on mammalian and yeast sequences. The extent to which these identified TbACC phosphosites are involved in the response to environmental lipids remains to be determined. Future phosphoproteomic analysis of TbACC will be required to determine if the phosphorylation of S5, S1999, and/or S2001 increases under high-lipid conditions or if other phosphosites are involved.

10.1128/mSphere.00164-18.5 FIG S4  Comparison of phosphorylation sites in human, yeast, and T. brucei ACCs. (A) Diagram drawn in scale comparing the protein domain organization and phosphorylation sites of human ACC1 (hsACC1), Saccharomyces cerevisiae ACC1 (ScACC1), and T. brucei ACC (TbACC). Biotin carboxylase domains (BC) are indicated in dark blue, biotin carboxyl carrier protein domains (BCCP) are indicated in yellow, and the carboxyltransferase domains (CT) are indicated in light blue. Phosphorylation sites are indicated with red vertical lines. The amino acid number of the site and the kinase(s) that is known to phosphorylate that residue (7, 8, 12, 16, 17) are indicated above each site. *, phosphorylation at this site does not alter enzyme activity. (B) ClustalW alignment of amino acid sequences of TbACC, ScACC1, and HsACC1 in the regions surrounding the phosphorylation sites indicated in panel A. Identical residues are shaded in black, and conserved residues are shaded in gray. Dashes indicate alignment gaps. Phosphorylated residues are boxed in red and indicated by an asterisk (*) above the alignment. Download FIG S4, TIF file, 2.9 MB. Copyright © 2018 Ray et al. 2018 Ray et al. This content is distributed under the terms of the Creative Commons Attribution 4.0 International license.

10.1128/mSphere.00164-18.7 TABLE S1  *In silico* prediction of TbACC phosphorylation sites. Download TABLE S1, DOCX file, 0.1 MB. Copyright © 2018 Ray et al. 2018 Ray et al. This content is distributed under the terms of the Creative Commons Attribution 4.0 International license.

Although the identified TbACC phosphosites are not well conserved, the regulatory impact of TbACC phosphorylation is consistent with the results seen with other ACCs, in that phosphorylation inhibits TbACC activity. As the primary ACC kinases in other organisms (12), AMPK and protein kinase A (PKA) represent potential candidates for the TbACC kinase(s) operating in this environmental lipid response pathway. Three observations support the idea of a role for AMPK in particular. First, exposure of bovine hepatocytes to fatty acids induced AMPK signaling and ACC phosphorylation (37), analogous to the pathway that may be operating in T. brucei. Second, treatment of BF lysates with EGCG, a known AMPK activator, stimulated TbACC phosphorylation (21), though it is unclear whether the effect on TbACC phosphorylation was direct or indirect, as EGCG has multiple cellular targets (38). Third, AMPK is already known to act in other environmental signaling pathways in T. brucei: in BF, quorum sensing, which triggers differentiation to short stumpy BFs by Stumpy Inducing Factor (39), and in PF, glucose sensing and regulation of procyclin expression (40). Interestingly, none of the proteomically identified phosphosites and only a few predicted phosphosites matched AMPK (20) (Table S1), though the prediction algorithms likely have limited utility in T. brucei, as mentioned above. Thus, it is possible that kinases other than AMPK or PKA are involved in TbACC regulation in response to environmental lipids. Further work will be required to confirm the identity of the TbACC kinase(s) in this environmental response pathway.

**FIG 5:**
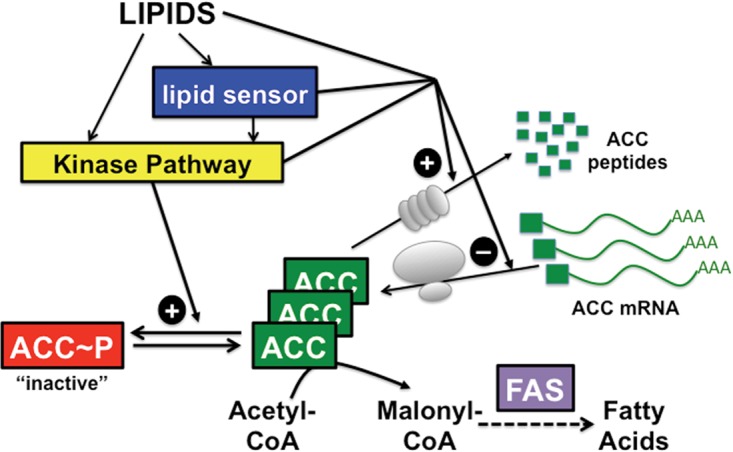
Current model for how environmental lipids modulate TbACC. Environmental lipids activate a kinase signaling pathway that leads to the phosphorylation and inactivation of TbACC. Environmental lipids also increase TbACC protein expression through repression of TbACC mRNA translation and/or stimulation of protein degradation. Lipids may regulate TbACC activity and expression directly or through the action of a lipid sensor. In addition, lipids may coordinately control TbACC protein levels and activity via the same kinase signaling pathway, or TbACC protein and activity may be regulated by distinct mechanisms. FAS, fatty acid synthesis.

Our current model for the regulation of TbACC activity by environmental lipids (Fig. 5) proposes that high levels of exogenous lipids activate a kinase pathway that phosphorylates and inhibits TbACC. This kinase pathway may be activated by direct lipid binding to a kinase or indirectly through a lipid sensor that transduces the lipid signal to the kinase pathway. At the same time, if exogenous lipids exceed a threshold, they also repress production of TbACC protein by inhibiting TbACC mRNA translation and/or promoting TbACC protein degradation. Thus, high-lipid conditions lead to lowered TbACC activity and reduced flux of acetyl-CoA into the fatty acid synthesis pathway. This TbACC downregulation enables PFs to reduce fatty acid synthesis when environmental lipids are abundant, helping to conserve carbon and energy for other metabolic processes. When levels of exogenous lipids become limited, the lipid sensor/kinase pathway is no longer activated, leaving the cellular phosphatases unopposed, which leads to dephosphorylation and activation of TbACC. If environmental lipids fall below the threshold, TbACC mRNA translation is derepressed and/or TbACC protein degradation is reduced, leading to increased TbACC protein levels. Overall, these changes in signaling result in increased TbACC activity and malonyl-CoA production for fatty acid synthesis, which enables the PFs to compensate for low lipid availability in their host environment.

The model described above suggests that TbACC phosphorylation and protein expression are modulated by lipids using a shared regulatory mechanism. However, it is also possible that TbACC phosphorylation and protein expression are regulated by lipids via independent mechanisms. Also, in this study, the observed changes in TbACC protein levels and in phosphorylation were each sufficient to account for the observed changes in TbACC activity, so it remains to be determined how each mode of regulation contributes to overall TbACC activity. One possibility is that modulating both TbACC protein levels and phosphorylation enables both a short-term response (phosphorylation) and a long-term response (protein levels) to environmental changes in lipids. Determining how T. brucei PFs detect exogenous lipids and how that information is transduced to the machinery controlling TbACC protein expression and phosphorylation and identifying the components of this environmental response pathway will be key to understanding how T. brucei senses its host environment and adapts its lipid metabolism to minimize energy expenditure, maximize use of host resources, and best take advantage of its parasitic lifestyle.

## MATERIALS AND METHODS

### Reagents and T. brucei strains.

All chemicals and reagents were purchased from Thermo Fisher Scientific Company and Sigma, except Iscove’s modified Dulbecco’s medium (IMDM) and minimum essential medium (MEM) were from Invitrogen; Serum Plus was from JRH Biosciences; and delipidated FBS was from Cocalico Biologicals. T. brucei strain 427 (wild-type PF and BF) was a kind gift from Paul Englund (Johns Hopkins School of Medicine). The PF and BF TbACC-myc cell lines, which possess one genomic locus of TbACC fused with a C-terminal c-myc epitope tag, were generated previously (15, 21).

### Growth media.

Serum is the only source of lipids in T. brucei growth media. Three different sera were used to prepare media: FBS, delipidated FBS (dFBS), and Serum Plus (SP) (Table 1). According to the manufacturers, both dFBS and SP have 20% of the lipids found in FBS. The normal growth media for PF T. brucei is SDM-79 (41) supplemented with heat-inactivated 10% FBS, resulting in 10% total serum lipids. For BF T. brucei, the normal growth media is HMI-9 (42) supplemented with heat-inactivated 10% FBS and 10% SP, resulting in 12% total serum lipids. To prepare low-lipid media for BFs and PFs, we replaced the 10% FBS component with heat-inactivated 10% dFBS, which resulted in final concentrations of 4% and 2% total serum lipids in BF and PF low-lipid media, respectively. High-lipid media for BFs and PFs was prepared by doubling the heat-inactivated 10% FBS component, resulting in final concentrations of 20% and 22% total serum lipids for BF and PF high-lipid media, respectively. See Table 1 for all media formulations. To generate low-, normal-, and high-lipid low-phosphate media, Cunningham’s phosphate-free media (43) was supplemented with dialyzed FBS as outlined in Table 1; 10% and 20% dialyzed FBS (Thermo Fisher) was used to generate the normal- and high-lipid Cunningham’s media, and 10% dialyzed delipidated FBS was used to generate the low-lipid Cunningham’s media. Dialyzed delipidated FBS was prepared by loading heat-inactivated delipidated FBS into dialysis tubing (10-kDa molecular weight cutoff) and dialyzing against three changes of 10 volumes of 150 mM NaCl for 6 to 10 h/change at 4°C. The dialyzed serum was clarified by centrifugation at 35,000 × *g* for 30 min at 4°C, subjected to filter sterilization (0.22-µm-pore-size filter), and stored at −20°C.

### Lysate preparation.

Hypotonic lysates were prepared from 0.5 ×10^9^ to 1 ×10^9^ cells as previously described (8, 44, 45). Hypotonic lysates were sheared by passage through a 27.5-gauge needle three times, and unbroken cells and debris were removed by centrifugation at 4°C at 1,000 × *g* for 10 min. The resulting supernatant was then clarified by centrifugation at 4°C at 16,000 × *g* for 10 min to yield the final hypotonic lysate. Radioimmunoprecipitation assay (RIPA) lysates were prepared from 1 × 10^9^ cells/ml as previously described (15) and were clarified by centrifugation at 4°C at 16,000 × *g* for 30 min. For both lysis methods, a protease inhibitor cocktail (final concentrations of 1 µg/ml leupeptin and 0.1 mM tosyl-l-lysine chloromethyl ketone hydrochloride [TLCK]) and a phosphatase inhibitor cocktail (HALT; Thermo Fisher) (1× final concentration) were added to the cell suspensions just prior to the lysis step.

### Western blotting.

Proteins were resolved by the use of SDS-PAGE (10% polyacrylamide resolving gel with a 4% polyacrylamide stacking gel) and transferred to a nitrocellulose membrane. All manipulations were carried out at room temperature. Appropriately exposed blots were analyzed by densitometry using ImageJ software (NIH) and values normalized to the indicated controls. Blots were probed for native TbACC using SA-HRP (Thermo Pierce), which recognizes the biotin prosthetic group of ACCs (15, 17). Membranes were blocked in streptavidin blocking buffer (1× Tris-buffered saline [TBS], 2.5% [wt/vol] nonfat dry milk, 0.05% [vol/vol] Tween 20) for ≥1 h followed by three 5-min washes in streptavidin wash buffer (1× TBS, 0.2% [wt/vol] nonfat dry milk, 0.05% [vol/vol] Tween 20). Blots were probed with SA-HRP for ≥2 h (1:200 dilution in streptavidin wash buffer). The blots were then washed twice with streptavidin wash buffer and twice with 1× TBS–0.05% (vol/vol) Tween 20 before chemiluminescent development was performed with Super Signal West Pico chemiluminescent substrate (Thermo Scientific). For anti-tubulin and anti-myc blotting, membranes were incubated in blocking buffer (1× TBS, 5% nonfat dry milk, 0.05% [vol/vol] Tween 20) for ≥1 h and then probed for ≥1 h with mouse monoclonal anti-tubulin antibody (clone B-5-1-2, ascites fluid; Sigma) (1:50,000) or mouse monoclonal anti-c-myc antibody (sc40; Santa Cruz Biotechnology) (1:250) diluted in blocking buffer. The blots were washed two to three times in blocking buffer and probed with peroxidase-conjugated polyclonal goat anti-mouse IgG (catalog no. 610-1302; Rockland) (1:10,000) in blocking buffer. Blots were then washed twice with blocking buffer and twice with 1× TBS–0.05% (vol/vol) Tween 20 before chemiluminescent development. TbACC protein levels were normalized to the tubulin loading control and then normalized to the normal-lipid condition. The statistical significance of differences in normalized data was assessed using the two-tailed Student’s *t* test.

### ACC activity assay.

PF and BF cells were grown to the late-log stage (for PF, 1 × 10^7^ to 2.5 × 10^7^ cells/ml; for BF, 2.5 × 10^6^ to 5 × 10^6^ cells/ml) under low-, normal-, and high-lipid media conditions. Hypotonic lysates were prepared as described above. To remove endogenous ATP and CoA substrates, lysates were fractionated through 1.5 to 2 ml G-50 Sephadex columns (Sigma) equilibrated in biotin carboxylase buffer (50 mM Tris-Cl [pH 8.0], 5 mM MgCl_2_, 5 mM dithiothreitol [DTT]) supplemented with 1× HALT phosphatase inhibitor cocktail. The protein concentrations of the chromatographed lysates were determined using a Bradford protein assay kit (Bio-Rad) with bovine serum albumin (BSA) as a standard, and samples were adjusted so that all lysates had the same protein concentration. A 4-step 2-fold dilution series (5 concentrations in total) of the chromatographed lysates was prepared and assayed for ACC activity as previously described (15). Briefly, chromatographed lysates were incubated in biotin carboxylase buffer with ATP, MgCl_2_, acetyl-CoA, and [^14^C]HCO_3_^−^ for 30 min at 30°C. The resulting [^14^C]malonyl-CoA product was subjected to acid precipitation, collected onto Whatman filters, and measured by scintillation counting. T. brucei ACC enzyme activity is labile, leading to variability in the levels of activity between lysate preparations (15). Thus, TbACC activity was first normalized to the no-ATP control activity and then expressed as a ratio normalized to the TbACC activity in normal-lipid media. The TbACC activity (measured in femtomoles of [^14^C]malonyl-CoA formed per 1 × 10^6^ cell equivalents) of the normal-media lysate preparations of all three independent experiments is indicated in Table S2 in the supplemental material. The statistical significance of differences in normalized data was assessed using the two-tailed Student’s *t* test.

10.1128/mSphere.00164-18.8 TABLE S2  ACC enzyme activity of control lysate preparations. Download TABLE S2, DOCX file, 0.1 MB. Copyright © 2018 Ray et al. 2018 Ray et al. This content is distributed under the terms of the Creative Commons Attribution 4.0 International license.

### TbACC-myc immunoprecipitation.

TbACC-myc was purified from hypotonic PF and BF lysates using a Pierce ProFound c-Myc-Tag immunoprecipitation/coimmunoprecipitation (IP/Co-IP) kit (Thermo Fisher). The manufacturer’s protocol was modified to obtain maximum yield as follows. Hypotonic lysates (500 µl or 5 × 10^8^ cell equivalents) were incubated with 10 to 15 µl of anti-myc agarose beads overnight at 4°C with constant end-over-end mixing. These conditions were sufficient to saturate the beads (see Fig. S5 in the supplemental material). The lysate/bead mixtures were loaded into empty spin columns and unbound proteins removed with three washes of 1× TBS–0.5% (vol/vol) Tween 20. Bound TbACC-myc was eluted using 150 mM glycine (pH 2.8) added 10 µl at a time. Total elution volumes were 100 µl and 60 µl for PF and BF TbACC-myc, respectively. Eluted TbACC-myc was immediately neutralized to a final pH of ~7.5 by the addition of 20 μl (PF) and 10 µl (BF) 100 mM Tris-Cl (pH 9.5).

10.1128/mSphere.00164-18.6 FIG S5  Estimation of ACC bound to anti-myc agarose beads. PF ACC-myc cells were grown in normal media to the mid-log phase (~3 days). ACC-myc was immunoprecipitated from 300 µl lysates (1.5 × 10^8^ cell equivalents) by incubation with 15 µl of a 25% slurry of anti-myc agarose beads as described in Materials and Methods. Samples of beads with bound ACC (0.4% of input), supernatant containing unbound fraction (0.1% of input), and eluted ACC-myc (48% of input) were resolved by the use of SDS-PAGE and transferred to nitrocellulose, and total ACC levels were detected by SA-HRP blotting and chemiluminescence. ACC levels in each fraction were determined by densitometry analysis of appropriately exposed films using Fiji software and comparison of the ACC signal in each fraction to a standard curve of total lysate (1.25 to 10 µg) loaded on the same gel. A representative blot of three independent experiments is shown. Download FIG S5, TIF file, 0.4 MB. Copyright © 2018 Ray et al. 2018 Ray et al. This content is distributed under the terms of the Creative Commons Attribution 4.0 International license.

### Metabolic ^32^P labeling of TbACC-myc.

The [^32^P]orthophosphate labeling was performed in low-phosphate media to ensure maximal ^32^P incorporation. PF ACC-myc cells were grown in normal-lipid SDM-79 media to the late-log phase (1 × 10^7^ to 2.5 × 10^7^ cells/ml). The cells were harvested by centrifugation (900 × *g*, 10 min, 25°C), washed thrice in Cunningham’s phosphate-free normal-lipid media, and resuspended to a final concentration of 0.5 × 10^8^ cells/ml in low-, normal-, or high-lipid Cunningham’s media. Cells were then incubated with 1 to 2 mCi [^32^P]orthophosphate (ARP103; American Radiolabeled Chemicals) (350 mCi/ml) for 16 h at 28°C/ 5% CO_2_. After labeling, TbACC-myc immunoprecipitates were prepared from hypotonic lysates as described above, resolved by the use of SDS-PAGE, and transferred to nitrocellulose. To assess [^32^P]orthophosphate labeling of TbACC-myc, the blot was exposed to X-ray film at room temperature. After the radioactivity decayed to undetectable levels, the same blot was probed for total TbACC as a loading control by SA-HRP blotting as described above. An appropriately exposed autoradiograph and blot were quantified by densitometry (ImageJ, NIH). Phosphorylated TbACC-myc values were normalized to total TbACC control values and then normalized to the normal-lipid condition.

### Phosphoprotein gel staining.

TbACC-myc immunoprecipitates prepared from PF TbACC-myc cells grown in low-, normal-, and high-lipid media were resolved by the use of SDS-PAGE, and the gels were processed and stained with Pro-Q Diamond phosphoprotein gel stain (Thermo Fisher) according to the manufacturer’s directions. Phosphostained gels were imaged under UV using a Gel Doc XR imaging system (Bio-Rad). An identically loaded nonstained gel prepared and run in parallel was transferred to nitrocellulose and probed with SA-HRP to detect total TbACC loaded. Appropriately exposed gel images and blots were analyzed by densitometry (ImageJ, NIH). Values for phosphorylated TbACC-myc were normalized to total TbACC loaded and then normalized to the normal-lipid condition.

### Lambda phosphatase treatment.

PF TbACC-myc cells were grown to late-log phase in normal-lipid media. Hypotonic lysates were prepared and incubated with anti-myc agarose beads for 16 h at 4°C with constant end-to-end mixing. TbACC-myc bound beads were washed five times by centrifugation (3,000 × *g*, 10 min) with 500-µl biotin carboxylase buffer. On the last wash, the beads were divided into two aliquots and resuspended in 200 µl Lambda phosphatase buffer alone (mock treatment) or buffer supplemented with 400 U of Lambda protein phosphatase (New England Biolabs) and incubated for 30 min at 30°C. Phosphatase-treated and mock-treated TbACC-myc bound beads were then washed twice in 100 µl biotin carboxylase buffer and assayed for ACC activity as described above by adding assay components directly to TbACC-myc bound beads. TbACC activity was first normalized to the no-ATP control and then expressed as a ratio normalized to the TbACC activity in the no-PPase control. The TbACC activity (measured in femtomoles [^14^C]malonyl-CoA formed per 1 × 10^6^ cell equivalents) of the no-PPase control in all three independent experiments is indicated in Table S2. The statistical significance of differences in normalized data was assessed using a two-tailed Student’s *t* test. To estimate the lysate equivalents of TbACC bound to the anti-myc beads, we compared results of control TbACC-myc pulldown experiments to a lysate standard curve by SA-HRP blotting (Fig. S5). These conditions saturated the beads, with an estimated 4.6 × 10^6^ cell equivalents (which corresponded to 43 ± 8.7 µg lysate equivalents) of TbACC bound per microliter of anti-myc beads. To confirm dephosphorylation, TbACC-myc immunoprecipitates were subjected to mock treatment and phosphatase treatment as described above and resolved by the use of SDS-PAGE, and the gel was stained with Pro-Q Diamond phosphoprotein stain. In parallel, an identically loaded nonstained gel was transferred to nitrocellulose and probed with SA-HRP to detect total levels of TbACC loaded. Due to the high background levels and narrow bandwidth, the gel and blot images were identically and uniformly processed by increasing the contrast and elongating the image in the *y* dimension to make the stained band easier to see. The original phosphoprotein-stained gel image without contrast and *y* aspect modification is included in Fig. S3.

## References

[1] Roheim PS, Carey M, Forte T, Vega GL 1979 Apolipoproteins in human cerebrospinal fluid. Proc Natl Acad Sci U S A 76:4646–4649. doi:10.1073/pnas.76.9.4646. 291993 PMC411636

[2] Trindade S, Rijo-Ferreira F, Carvalho T, Pinto-Neves D, Guegan F, Aresta-Branco F, Bento F, Young SA, Pinto A, Van Den Abbeele J, Ribeiro RM, Dias S, Smith TK, Figueiredo LM 2016 *Trypanosoma brucei* parasites occupy and functionally adapt to the adipose tissue in mice. Cell Host Microbe 19:837–848. doi:10.1016/j.chom.2016.05.002. 27237364 PMC4906371

[3] Bringaud F, Rivière L, Coustou V 2006 Energy metabolism of trypanosomatids: adaptation to available carbon sources. Mol Biochem Parasitol 149:1–9. doi:10.1016/j.molbiopara.2006.03.017. 16682088

[4] Dyer NA, Rose C, Ejeh NO, Acosta-Serrano A 2013 Flying tryps: survival and maturation of trypanosomes in tsetse flies. Trends Parasitol 29:188–196. doi:10.1016/j.pt.2013.02.003. 23507033

[5] Field MC, Lumb JH, Adung’a VO, Jones NG, Engstler M 2009 Macromolecular trafficking and immune evasion in African trypanosomes. Int Rev Cell Mol Biol 278:1–67. doi:10.1016/S1937-6448(09)78001-3. 19815176

[6] Mellors A, Samad A 1989 The acquisition of lipids by African trypanosomes. Parasitol Today 5:239–244. doi:10.1016/0169-4758(89)90255-X. 15463224

[7] Uttaro AD 2014 Acquisition and biosynthesis of saturated and unsaturated fatty acids by trypanosomatids. Mol Biochem Parasitol 196:61–70. doi:10.1016/j.molbiopara.2014.04.001. 24726787

[8] Morita YS, Paul KS, Englund PT 2000 Specialized fatty acid synthesis in African trypanosomes: myristate for GPI anchors. Science 288:140–143. doi:10.1126/science.288.5463.140. 10753118

[9] Lee SH, Stephens JL, Paul KS, Englund PT 2006 Fatty acid synthesis by elongases in trypanosomes. Cell 126:691–699. doi:10.1016/j.cell.2006.06.045. 16923389

[10] Stephens JL, Lee SH, Paul KS, Englund PT 2007 Mitochondrial fatty acid synthesis in *Trypanosoma brucei*. J Biol Chem 282:4427–4436. doi:10.1074/jbc.M609037200. 17166831

[11] Tong L 2013 Structure and function of biotin-dependent carboxylases. Cell Mol Life Sci 70:863–891. doi:10.1007/s00018-012-1096-0. 22869039 PMC3508090

[12] Brownsey RW, Boone AN, Elliott JE, Kulpa JE, Lee WM 2006 Regulation of acetyl-CoA carboxylase. Biochem Soc Trans 34:223–227. doi:10.1042/BST20060223. 16545081

[13] Hardie DG, Schaffer BE, Brunet A 2016 AMPK: an energy-sensing pathway with multiple inputs and outputs. Trends Cell Biol 26:190–201. doi:10.1016/j.tcb.2015.10.013. 26616193 PMC5881568

[14] Winder WW, Thomson DM 2007 Cellular energy sensing and signaling by AMP-activated protein kinase. Cell Biochem Biophys 47:332–347. doi:10.1007/s12013-007-0008-7. 17652779

[15] Vigueira PA, Paul KS 2011 Requirement for acetyl-CoA carboxylase in *Trypanosoma brucei* is dependent upon the growth environment. Mol Microbiol 80:117–132. doi:10.1111/j.1365-2958.2011.07563.x. 21306439 PMC3656591

[16] Doering TL, Pessin MS, Hoff EF, Hart GW, Raben DM, Englund PT 1993 Trypanosome metabolism of myristate, the fatty acid required for the variant surface glycoprotein membrane anchor. J Biol Chem 268:9215–9222. 8486622

[17] Nikolau BJ, Wurtele ES, Stumpf PK 1985 Use of streptavidin to detect biotin-containing proteins in plants. Anal Biochem 149:448–453. doi:10.1016/0003-2697(85)90596-2. 2866733

[18] Haneji T, Koide SS 1989 Transblot identification of biotin-containing proteins in rat liver. Anal Biochem 177:57–61. doi:10.1016/0003-2697(89)90013-4. 2742154

[19] Edelstein C 1986 General properties of plasma lipoproteins and apolipoproteins, p 495–505. *In* Scanu AM, Spector AA (ed), Biochemistry and biology of plasma lipoproteins. Marcel Dekker, New York, NY.

[20] Urbaniak MD, Martin DM, Ferguson MA 2013 Global quantitative SILAC phosphoproteomics reveals differential phosphorylation is widespread between the procyclic and bloodstream form lifecycle stages of *Trypanosoma brucei*. J Proteome Res 12:2233–2244. doi:10.1021/pr400086y. 23485197 PMC3646404

[21] Vigueira PA, Ray SS, Martin BA, Ligon MM, Paul KS 2012 Effects of the green tea catechin (-)-epigallocatechin gallate on *Trypanosoma brucei*. Int J Parasitol Drugs Drug Resist 2:225–229. doi:10.1016/j.ijpddr.2012.09.001. 24533284 PMC3862400

[22] Tong L 2005 Acetyl-coenzyme A carboxylase: crucial metabolic enzyme and attractive target for drug discovery. Cell Mol Life Sci 62:1784–1803. doi:10.1007/s00018-005-5121-4. 15968460 PMC11139103

[23] Siegel TN, Gunasekera K, Cross GA, Ochsenreiter T 2011 Gene expression in *Trypanosoma brucei*: lessons from high-throughput RNA sequencing. Trends Parasitol 27:434–441. doi:10.1016/j.pt.2011.05.006. 21737348 PMC3178736

[24] Schwede A, Kramer S, Carrington M 2012 How do trypanosomes change gene expression in response to the environment? Protoplasma 249:223–238. doi:10.1007/s00709-011-0282-5. 21594757 PMC3305869

[25] Kramer S 2012 Developmental regulation of gene expression in the absence of transcriptional control: the case of kinetoplastids. Mol Biochem Parasitol 181:61–72. doi:10.1016/j.molbiopara.2011.10.002. 22019385

[26] Yoon S, Lee MY, Park SW, Moon JS, Koh YK, Ahn YH, Park BW, Kim KS 2007 Up-regulation of acetyl-CoA carboxylase alpha and fatty acid synthase by human epidermal growth factor receptor 2 at the translational level in breast cancer cells. J Biol Chem 282:26122–26131. doi:10.1074/jbc.M702854200. 17631500

[27] David M, Gabdank I, Ben-David M, Zilka A, Orr I, Barash D, Shapira M 2010 Preferential translation of Hsp83 in *Leishmania* requires a thermosensitive polypyrimidine-rich element in the 3′ UTR and involves scanning of the 5′ UTR. RNA 16:364–374. doi:10.1261/rna.1874710. 20040590 PMC2811665

[28] Walrad P, Paterou A, Acosta-Serrano A, Matthews KR 2009 Differential trypanosome surface coat regulation by a CCCH protein that co-associates with procyclin mRNA cis-elements. PLoS Pathog 5:e1000317. doi:10.1371/journal.ppat.1000317. 19247446 PMC2642730

[29] Li Z, Zou CB, Yao Y, Hoyt MA, McDonough S, Mackey ZB, Coffino P, Wang CC 2002 An easily dissociated 26 S proteasome catalyzes an essential ubiquitin-mediated protein degradation pathway in *Trypanosoma brucei*. J Biol Chem 277:15486–15498. doi:10.1074/jbc.M109029200. 11854272

[30] Minia I, Clayton C 2016 Regulating a post-transcriptional regulator: protein phosphorylation, degradation and translational blockage in control of the trypanosome stress-response RNA-binding protein ZC3H11. PLoS Pathog 12:e1005514. doi:10.1371/journal.ppat.1005514. 27002830 PMC4803223

[31] Qi L, Heredia JE, Altarejos JY, Screaton R, Goebel N, Niessen S, Macleod IX, Liew CW, Kulkarni RN, Bain J, Newgard C, Nelson M, Evans RM, Yates J, Montminy M 2006 TRB3 links the E3 ubiquitin ligase COP1 to lipid metabolism. Science 312:1763–1766. doi:10.1126/science.1123374. 16794074

[32] Ma J, Yan R, Zu X, Cheng JM, Rao K, Liao DF, Cao D 2008 Aldo-keto reductase family 1 B10 affects fatty acid synthesis by regulating the stability of acetyl-CoA carboxylase-alpha in breast cancer cells. J Biol Chem 283:3418–3423. doi:10.1074/jbc.M707650200. 18056116

[33] Nett IR, Martin DM, Miranda-Saavedra D, Lamont D, Barber JD, Mehlert A, Ferguson MA 2009 The phosphoproteome of bloodstream form *Trypanosoma brucei*, causative agent of African sleeping sickness. Mol Cell Proteomics 8:1527–1538. doi:10.1074/mcp.M800556-MCP200. 19346560 PMC2716717

[34] Hunkeler M, Stuttfeld E, Hagmann A, Imseng S, Maier T 2016 The dynamic organization of fungal acetyl-CoA carboxylase. Nat Commun 7:11196. doi:10.1038/ncomms11196. 27073141 PMC4833862

[35] Woods A, Munday MR, Scott J, Yang X, Carlson M, Carling D 1994 Yeast SNF1 is functionally related to mammalian AMP-activated protein kinase and regulates acetyl-CoA carboxylase *in vivo*. J Biol Chem 269:19509–19515. 7913470

[36] Witters LA, Watts TD 1990 Yeast acetyl-CoA carboxylase: *in vitro* phosphorylation by mammalian and yeast protein kinases. Biochem Biophys Res Commun 169:369–376. doi:10.1016/0006-291X(90)90341-J. 1972618

[37] Li X, Li X, Chen H, Lei L, Liu J, Guan Y, Liu Z, Zhang L, Yang W, Zhao C, Fu S, Li P, Liu G, Wang Z 2013 Non-esterified fatty acids activate the AMP-activated protein kinase signaling pathway to regulate lipid metabolism in bovine hepatocytes. Cell Biochem Biophys 67:1157–1169. doi:10.1007/s12013-013-9629-1. 23690240

[38] Wang S, Moustaid-Moussa N, Chen L, Mo H, Shastri A, Su R, Bapat P, Kwun I, Shen CL 2014 Novel insights of dietary polyphenols and obesity. J Nutr Biochem 25:1–18. doi:10.1016/j.jnutbio.2013.09.001. 24314860 PMC3926750

[39] Saldivia M, Ceballos-Pérez G, Bart JM, Navarro M 2016 The AMPKalpha1 pathway positively regulates the developmental transition from proliferation to quiescence in *Trypanosoma brucei*. Cell Rep 17:660–670. doi:10.1016/j.celrep.2016.09.041. 27732844 PMC5074416

[40] Clemmens CS, Morris MT, Lyda TA, Acosta-Serrano A, Morris JC 2009 *Trypanosoma brucei* AMP-activated kinase subunit homologs influence surface molecule expression. Exp Parasitol 123:250–257. doi:10.1016/j.exppara.2009.07.010. 19647733 PMC2774744

[41] Brun R, Schönenberger M 1979 Cultivation and *in vitro* cloning or procyclic culture forms of *Trypanosoma brucei* in a semi-defined medium. Acta Trop 36:289–292. 43092

[42] Hirumi H, Hirumi K 1989 Continuous cultivation of *Trypanosoma brucei* blood stream forms in a medium containing a low concentration of serum protein without feeder cell layers. J Parasitol 75:985–989. doi:10.2307/3282883. 2614608

[43] Kaminsky R, Beaudoin E, Cunningham I 1988 Cultivation of the life cycle stages of *Trypanosoma brucei* sspp. Acta Trop 45:33–43. 2896444

[44] Bangs JD, Uyetake L, Brickman MJ, Balber AE, Boothroyd JC 1993 Molecular cloning and cellular localization of a BiP homologue in *Trypanosoma brucei*. Divergent ER retention signals in a lower eukaryote. J Cell Sci 105:1101–1113. 8227199 10.1242/jcs.105.4.1101

[45] Roggy JL, Bangs JD 1999 Molecular cloning and biochemical characterization of a VCP homolog in African trypanosomes. Mol Biochem Parasitol 98:1–15. doi:10.1016/S0166-6851(98)00114-5. 10029305

