# Supplementary material for: Regulation of Trypanosoma brucei Acetyl Coenzyme A Carboxylase by Environmental Lipids
Source: mSphere. 2018 Jul 11;3(4):e00164-18. doi: 10.1128/mSphere.00164-18 (PMC6041502; doi:10.1128/mSphere.00164-18)
Supplement: TEXT S1 [file sph004182586s1.docx]

**Supplemental Information**

**Quantitative reverse transcriptase PCR (qRT-PCR).** BF and PF WT cells (3-5 x 10^6^ cells) were grown to mid-logarithmic stage (~5 x 10^5^ for BFs and ~5 x 10^6^ for PFs) and washed in bicine-buffered saline with glucose (BBSG; 50 mM Bicine-Na+, pH 8, 50 mM NaCl, 5 mM KCl, 70 mM glucose). Total RNA was extracted using the Aurum Total RNA Mini Kit (Bio-Rad) according to the manufacturer’s protocol, except final elution volume was 40 µl. 100-200 ng of total RNA was used to generate cDNA using the iScript cDNA Synthesis Kit (Bio-Rad) according to the manufacturer’s protocol. qRT-PCR was performed using a C1000 Touch Thermal Cycler equipped with a CFX96 Real-Time System (Bio-Rad) with SensiFAST SYBR & Fluorescein Mix (Bioline) in a final volume of 30 µl. The PCR reactions consisted of 2-4 µl of cDNA sample and 0.2 μM of each primer pair. *T. brucei* ACC-specific primers were designed using Primer3 software (1,2): the forward TbACC primer (5’-TTT CGT AAG TTG AAG TCT GG-3’) and reverse TbACC primer (5’-CTT AGT CGG ATC AAT GTC AC-3’) produced a 192-bp amplicon. Parallel reactions using actin-specific primers (a kind gift from Dr. Meredith Morris, Clemson University) were performed as the internal comparative control. The forward Actin primer (F Actin, 5’-GCC ACG TAT TTC CAT CCA TC-3’) and reverse Actin primer (R Actin, 5’-CCT GAG CTT CAT CAC CAA CA-3’) produced a 120-bp amplicon (3). Amplification conditions were as follows: 2 min. at 95°C followed by 40 cycles of 30 sec. at 95°C, 1 min. at 57°C, and 30 sec. at 72°C. The data were analyzed with Bio-Rad CFX Manager Software v3.1, and the comparative Ct method (ΔΔCt) was used to calculate the relative TbACC expression levels normalized to actin (4).

**Alignment of Acetyl-CoA Carboxylase sequences.** The complete amino acid sequences of TREU 927 *Trypanosoma brucei* ACC (TriTrypDB Gene ID: Tb927.8.7100), *Saccharomyces cerevisiae* ACC1 (NCBI Accession # AAA20073), and *Homo sapiens* ACC1 (Isoform 1, NCBI Accession # NP_942131) were down-loaded from the indicated databases, aligned using the ClustalW algorithm (5), and displayed using Boxshade (version 3.2.1, Expasy Bioinformatics Resource Portal, Swiss Institute of Bioinformatics [http://www.ch.embnet.org/software/BOX_form.html]) (Fig. S2A). The complete alignment was published previously (6). Known regulatory phosphorylation sites in human and yeast ACCs are indicated in red (7-11). TbACC phosphorylation sites identified by phosphoproteomics (12) are also indicated in red. The regions of the alignment surrounding each cluster of phosphorylation sites are shown in Fig. S2B.

***In silico* analysis of potential phosphorylation sites in TbACC.** To take an unbiased approach to identifying potential regulatory phosphosites, the predicted 2181 amino acid open-reading frame of TbACC (TriTrypDB Gene ID:Tb927.8.7100) was submitted to two phosphosite prediction algorithms: NetPhos 2.0, which predicts Ser, Thr, and Tyr phosphosites in eukaryotic proteins (13); and NetPhosYeast 1.0, which predicts Ser and Thr phosphosites in yeast proteins (14). Eight Ser and two Thr sites scored highly in both algorithms (≥0.90 by Netphos 2.0 and ≥0.5 by NetPhosYeast) (Table S1). Of the phosphosites identified by proteomics (12), only S2001 was also identified by the prediction algorithms.

To examine what kinases might potentially phosphorylate TbACC, the phosphosites in Table S1 were subjected to two kinase-specific prediction algorithms, KinasePhos 2.0 (15) and the GPS 3.0 web server for the prediction of protein post-translational modification sites [http://gps.biocuckoo.org] to assess candidate kinases for each identified phosphosite. The two kinase-specific algorithms predicted multiple high scoring candidate kinases for all sites (≥0.50 by KinasePhos and a ≥1.5 ration of GPS score to cut-off value), with those kinases scored highly by both algorithms indicated in red (Table S1).

Although PKA and AMPK-related sites, including the Ca^++^/calmodulin-dependent protein kinase-like kinases (CAMKL) were identified, most predicted AMPK and PKA sites had low or below-threshold confidence scores (data not shown). Instead, each site returned predictions for a variety of Ser/Thr kinases and non-receptor tyrosine kinases. Within the set of congruent Ser/Thr phosphosites predicted by both NetPhos and NetPhosYeast algorithms, the kinase-specific site predictions overlapped on only three Ser sites: S396 and S2001 (MAPK); and S1355 (Polo-like kinase (PLK)), though in each case the highest scoring kinase differed between the two algorithms (Table S1).

**REFERENCES**

1. **Untergasser A, Cutcutache I, Koressaar T, Ye J, Faircloth BC, Remm M, and Rozen SG.** 2012. Primer3--new capabilities and interfaces. Nucl Acids Res **40**, e115.

2. **Untergasser A, Nijveen H, Rao X, Bisseling T, Geurts R, and Leunissen JA.** 2007. Primer3Plus, an enhanced web interface to Primer3. Nucl Acids Res **35**, W71-74.

3. **Dodson H.** 2011. Regulation of *Trypanosoma brucei* hexokinase 1 and 2 on multiple levels: transcript abundance, protein expression and enzyme activity. PhD thesis. Clemson University, Clemson, SC.

4. **Livak KJ, Schmittgen TD.** 2001. Analysis of relative gene expression data using real-time quantitative PCR and the 2(-Delta Delta C(T)) Method. Methods **25**, 402-408.

5. **Larkin MA, Blackshields G, Brown NP, Chenna R, McGettigan PA, McWilliam H, Valentin F, Wallace IM, Wilm A, Lopez R, Thompson JD, Gibson TJ, Higgins DG.** 2007. Clustal W and Clustal X version 2.0. Bioinformatics **23**, 2947-2948.

6. **Vigueira PA, Paul KS.** 2011. Requirement for acetyl-CoA carboxylase in *Trypanosoma brucei* is dependent upon the growth environment. Mol Microbiol **80**, 117-132.

7. **Brownsey RW, Boone AN, Elliott JE, Kulpa JE, Lee WM.** 2006. Regulation of acetyl-CoA carboxylase. Biochem Soc Trans **34**, 223-227.

8. **Woods A, Munday MR, Scott J, Yang X, Carlson M, Carling D.** 1994. Yeast SNF1 is functionally related to mammalian AMP-activated protein kinase and regulates acetyl-CoA carboxylase *in vivo*. J Biol Chem **269**, 19509-19515.

9. **Pan DA, Hardie DG.** 2002. A homologue of AMP-activated protein kinase in *Drosophila melanogaster* is sensitive to AMP and is activated by ATP depletion. Biochem J **367**, 179-186.

10. **Savage LJ., Ohlrogge JB.** 1999. Phosphorylation of pea chloroplast acetyl-CoA carboxylase. Plant J **18**, 521-527.

11. **Zu X, Zhong J, Luo D, Tan J, Zhang Q, Wu Y, Liu J, Cao R, Wen G, Cao D.** 2013. Chemical genetics of acetyl-CoA carboxylases. Molecules **18**, 1704-1719.

12. **Urbaniak MD, Martin DM, Ferguson MA.** 2013. Global quantitative SILAC phosphoproteomics reveals differential phosphorylation is widespread between the procyclic and bloodstream form life cycle stages of *Trypanosoma* *brucei*. J Proteome Res **12**, 2233-2244.

13. **Blom N, Gammeltoft S, Brunak S.** 1999. Sequence and structure-based prediction of eukaryotic protein phosphorylation sites. J Mol Biol **294**, 1351-1362.

14. **Ingrell CR, Miller ML, Jensen ON, Blom N.** 2007. NetPhosYeast: prediction of protein phosphorylation sites in yeast. Bioinformatics **23**, 895-897.

15. **Wong YH, Lee TY, Liang HK, Huang CM, Wang TY, Yang YH, Chu CH, Huang HD, Ko MT, Hwang JK.** 2007. KinasePhos 2.0: a web server for identifying protein kinase-specific phosphorylation sites based on sequences and coupling patterns. Nucl Acids Res **35**, W588-594.
