# Supplementary figures and images for: Regulation of Trypanosoma brucei Acetyl Coenzyme A Carboxylase by Environmental Lipids
Source: mSphere. 2018 Jul 11;3(4):e00164-18. doi: 10.1128/mSphere.00164-18 (PMC6041502; doi:10.1128/mSphere.00164-18)

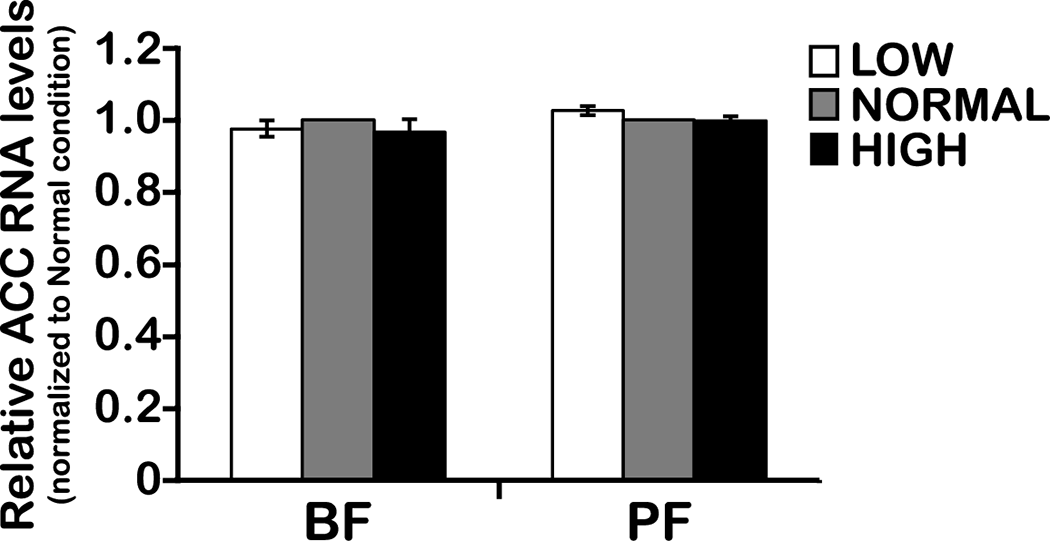

Supplement: FIG S1 [file sph004182586sf1.tif]

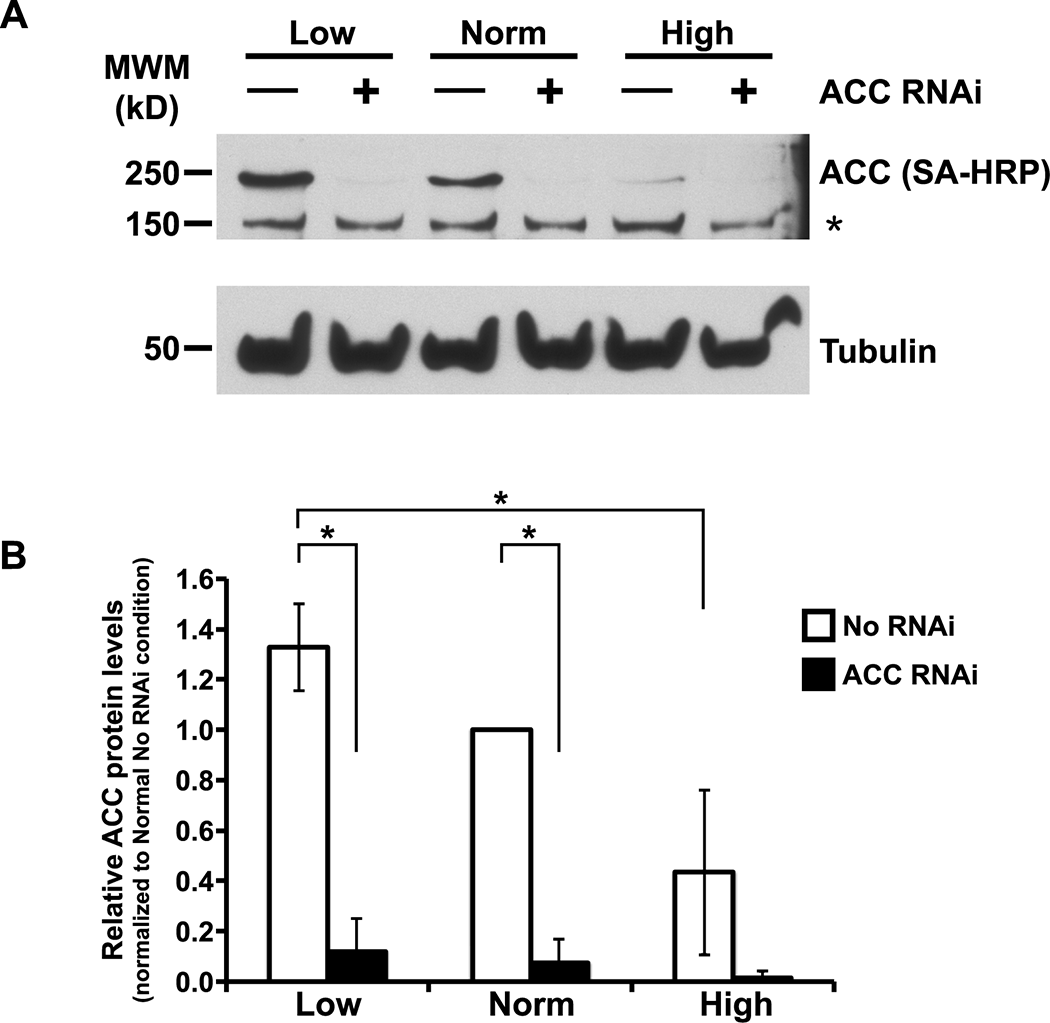

Supplement: FIG S2 [file sph004182586sf2.tif]

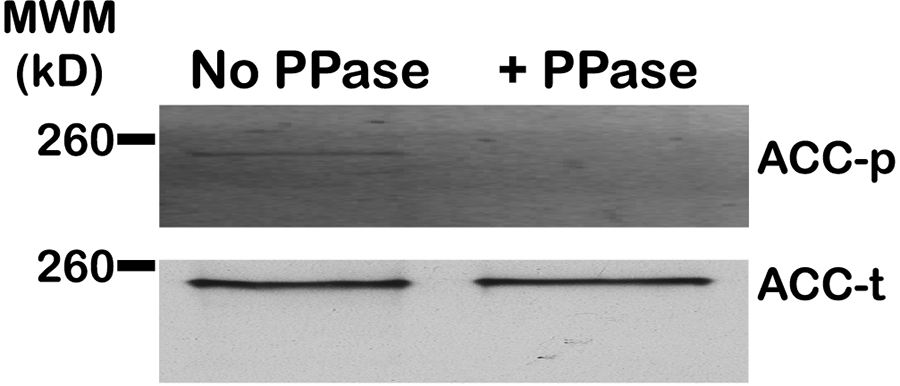

Supplement: FIG S3 [file sph004182586sf3.tif]

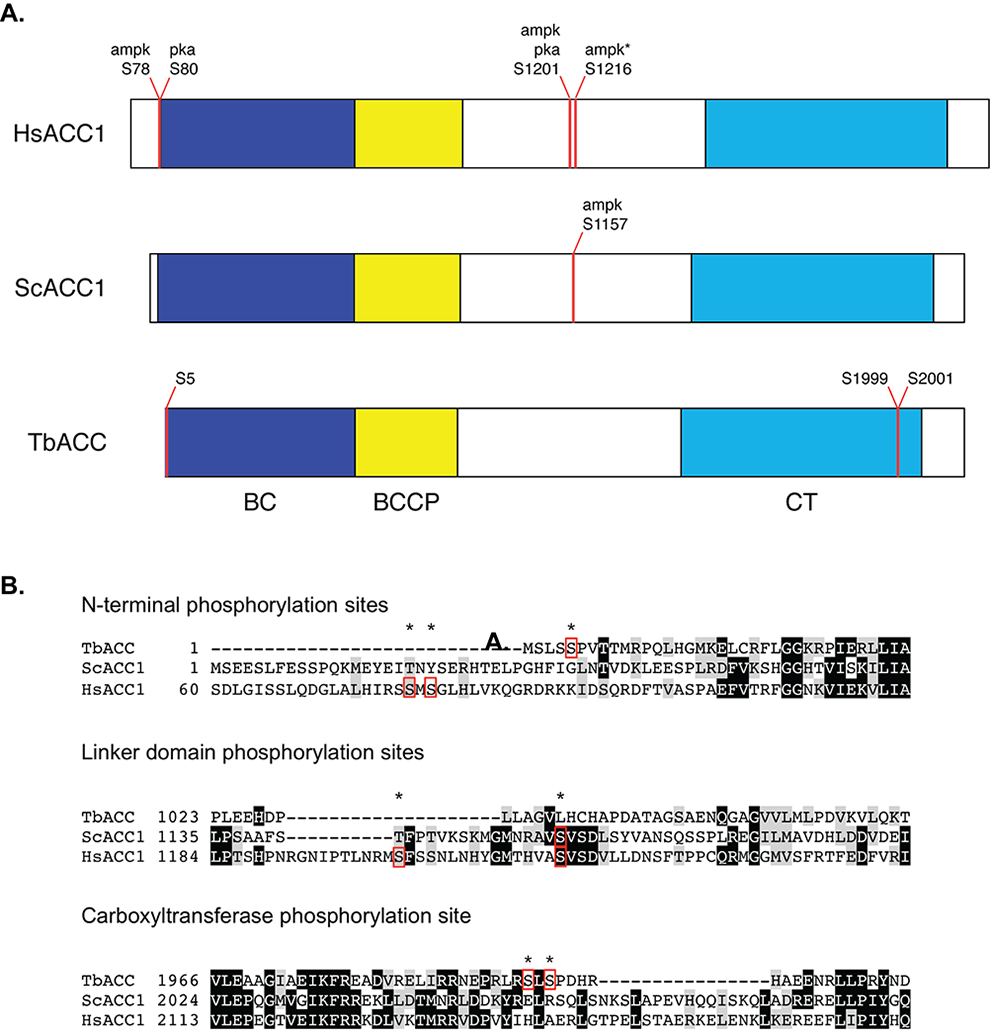

Supplement: FIG S4 [file sph004182586sf4.tif]

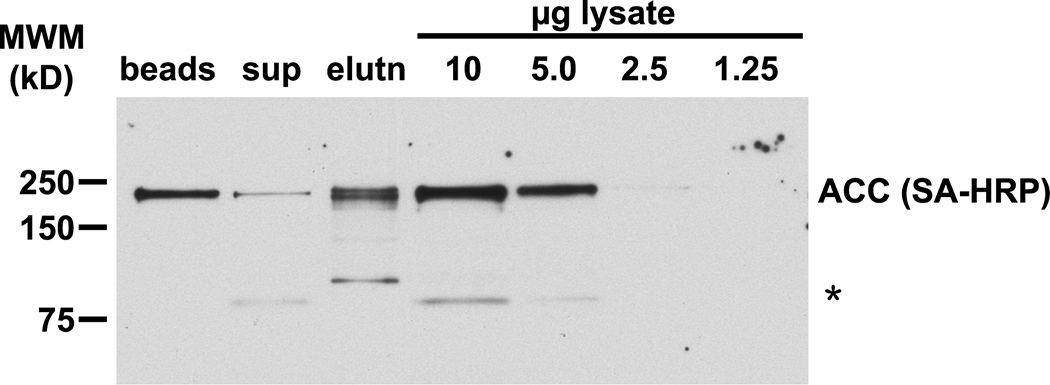

Supplement: FIG S5 [file sph004182586sf5.tif]
