# Supplementary material for: Regulation of Trypanosoma brucei Acetyl Coenzyme A Carboxylase by Environmental Lipids
Source: mSphere. 2018 Jul 11;3(4):e00164-18. doi: 10.1128/mSphere.00164-18 (PMC6041502; doi:10.1128/mSphere.00164-18)
Supplement: TABLE S1 [file sph004182586st1.docx]

**Table S1.** ***In silico* Prediction of TbACC Phosphorylation Sites**

| **Phosphosite prediction** | | | | **Kinase Prediction** | |  |
| --- | --- | --- | --- | --- | --- | --- |
| **AA#** | **Sequence** | **NetPhos 2.0**  **(Score ≥ 0.90)**^1^ | **NetPhos**  **Yeast 1.0**^2^  **(Score ≥ 0.50)** | **KinasePhos 2.0**  **(SVM Score ≥ 0.50)** | **GPS 3.0**  **High Scoring Kinases (Score/Cut-Off ≥ 1.5)** | **Phospho-proteomic Evidence** |
|  |  |  |  |  |  |  |
| 5 | MSLS**S**PVTT | 0.474 | 0.802 (yes) | ATM (0.983)*  CK1 (0.758)  PLK (0.722) | MAPK (6.158)  CDK (3.326)*  CK1 (3.174)  GSK3 (2.050)*  PLK (1.613) | Yes (43) |
| 197 | YEKA**Y**ISTA | 0.970 | n/a | all below threshold | TEC (1.525)^†^ |  |
| 396 | PYDT**S**PIDF | 0.918 | 0.677 (yes) | ATM (0.905)  CK2 (0.778)  MAPK (0.754) | MAPK (3.533)  CDK (3.193) |  |
| 585 | ESES**Y**VNRS | 0.985 | n/a | all below threshold | SYK (2.771)*  FAK (2.216)*  SRC (1.542)* |  |
| 600 | MGLT**S**PTEI | 0.995 | 0.683 (yes) | ATM (0.962)  CK2 (0.889) | MAPK (5.976)  CDK (3.193)* |  |
| 730 | VAEI**T**PDDP | 0.975 | 0.666 (yes) | all below threshold | MAPK (4.485)  CDK (2.773)  STE20/PAK (1.765)  GSK3 (1.662) |  |
| 766 | ERLD**S**LARA | 0.988 | 0.686 (yes) | ATM (0.877)*  AUR (0.796)*  PLK (0.752)  GSK3 (0.711) | AKT (2.619)*  STE20/PAK (2.549)  PLK (1.596)  PKC (1.506) |  |
| 1046 | ATAG**S**AENQ | 0.945 | 0.578 (yes) | PLK (0.858)*  ATM (0.772) | all below threshold |  |
| 1140 | PRTF**T**YRSA | 0.978 | 0.581 (yes) | all below threshold | PDHK (3.565)  CDK (3.117)  AKT (1.753)*  PKC (1.720)  CAMKL (1.62) |  |
| 1197 | KKSV**S**FLEH | 0.994 | 0.544 (yes) | ATM (0.940)  CK1 (0.875) | PDHK (3.984)  PKG (1.56)* |  |
| 1315 | VSSS**S**GASA | 0.909 | 0.519 (yes) | AUR (0.921)  ATM (0.909) | CDK (1.548) |  |
| 1355 | TSSD**S**VQEQ | 0.992 | 0.550 (yes) | ATM (0.987)*  PLK (0.769) | CK1 (1.818)*  PLK (1.663) |  |
| 1478 | ATEL**Y**LDPA | 0.955 | n/a | all below threshold | SRC (1.647)* |  |
| 1716 | GKEV**Y**SDNS | 0.952 | n/a | all below threshold | SRC (1.725)* |  |
| 1999 | PRLR**S**LSPD | 0.786 | 0.458 | AUR (0.943)*  ATM (0.888)  PAK (0.852)  PLK (0.814)* | STE20/PAK (3.017)  AKT (2.836)*  PKC (1.702) | Yes (43) |
| 2001 | LRSL**S**PDHR | 0.987 | 0.822 (yes) | ATM (0.811)  MAPK (0.804)  AUR (0.769) | MAPK (4.947)  CDK (2.708)  AKT (2.611)*  GSK3 (1.988)  PKA (1.65) | Yes (43) |

^1^Threshold value for inclusion indicated in parentheses

^2^NetPhos Yeast 1.0 only detects Ser/Thr phosphosites

n/a = not applicable (no search for Y)

* = detected in other kinase-specific search, but below our indicated threshold

Kinases indicated in red scored above threshold in both algorithm
