# Supplementary material for: Regulation of Trypanosoma brucei Acetyl Coenzyme A Carboxylase by Environmental Lipids
Source: mSphere. 2018 Jul 11;3(4):e00164-18. doi: 10.1128/mSphere.00164-18 (PMC6041502; doi:10.1128/mSphere.00164-18)
Supplement: TABLE S2 [file sph004182586st2.docx]

**Table S2.** **ACC Enzyme Activity of Control Lysate Preparations**

|  | **Trial 1** | **Trial 2** | **Trial 3** | **Average ± S.D.** |
| --- | --- | --- | --- | --- |
| **Fig. 1A** |  |  |  |  |
| BF Normal Media | 0.71 | 0.79 | 1.40 | 0.96 ± 0.38 |
| PF Normal Media | 9.44 | 20.66 | 34.23 | 21.44 ± 12.41 |
| **Fig. 1B** |  |  |  |  |
| PF Low Lipid | 2.47 | 6.39 | 11.04 | 6.63 ± 4.29 |
| **Fig. 4A** |  |  |  |  |
| PF Low, No PPase | 2.56 | 4.23 | 5.70 | 4.16 ± 1.57 |

ACC enzyme activity expressed in fmoles of [^14^C]malonyl-CoA generated per 1 x 10^6^ cell equivalents in 30 min. at 30°C.
